# A simple method for in vitro preparation of natural killer cells from cord blood

**DOI:** 10.1186/s12896-019-0564-0

**Published:** 2019-11-21

**Authors:** Yong Xu Mu, Yu Xia Zhao, Bing Yao Li, Hong Jing Bao, Hui Jiang, Xiao Lei Qi, Li Yun Bai, Yun Hong Wang, Zhi Jie Ma, Xiao Yun Wu

**Affiliations:** 1 Interventional Department, the First Affiliated Hospital of Baotou Medical College, Inner Mongolia University of Science and Technology, Baotou, Inner Mongolia China; 2 Department of Blood, the People’s Hospital of Xing’an League, Xing’an League, Inner Mongolia China; 3 Department of Medicine, Chifeng Cancer Hospital, Chifeng, Inner Mongolia China; 4 Department of Technology, Stem Cell Medicine Engineering & Technology Research Center of Inner Mongolia, Huhhot, Inner Mongolia China; 5 Department of Research and Development, Beijing Jingmeng Stem Cell Technology CO., LTD, Beijing, China; 6 Department of Pharmacy, Beijing Friendship Hospital, Capital Medical University, Beijing, China

**Keywords:** Cord blood, Natural killer cells, Expansion, Cytotoxicity, Immunotherapy

## Abstract

**Background:**

Cord Blood (CB) has been considered a promising source of natural killer (NK) cells for cellular immunotherapy. However, it is difficult to expand the large numbers of highly pure NK cells from CB without cell sorting and feeder cells/multiple cytokines. In this study, we try to develop a simple, safe and economical method for ex vivo expansion and purification of NK cells from CB without cell sorting and feeder cells/multiple cytokines.

**Results:**

The large numbers (mean: 1.59 × 10^10^) of highly pure (≥90%) NK cells from CB could be obtained through interleukin-2, group A streptococcus and zoledronate stimulation of mononuclear cells using the 21-day culture approach. When compared to resting NK cells, expanded NK cells were a higher expression of activating receptors CD16, NKG2D, NKp30, NKp44, NKp46 and activating markers CD62L and CD69, while the inhibitory receptors, CD158a and CD158b remained largely unchanged. In addition, these cells showed a higher concentration of IFN-γ, TNF-α and GM-CSF secretion and cytotoxicity to K562 cells and acute myeloid leukemia targets than resting NK cells.

**Conclusion:**

We develop a simple, safe and economical method to obtain high yield, purity, and functionality NK cells from CB without cell sorting and feeder cells/multiple cytokines.

## Background

Allogeneic natural killer (NK) cell infusion is promising for cancer immunotherapy because of the “missing self” hypothesis [1]. Cord blood (CB), serves as an immediate “off-the-shelf” source of NK cells, has been considered an attractive source of allogeneic NK cells for therapeutic infusion [2, 3]. However, a major challenge of cell therapy with NK cells is to attain sufficient amount of highly pure cells (> 70% pure, > 1 × 10^9^) because of the low frequency and number (<20% pure, <1 × 10^8^) of NK cells in the CB [3, 4]. To provide allogeneic NK cells with high yield, purity and functionality, some methods have been developed to purify and expand NK cells from CB ex vivo [5–10].

To date, most methods for in vitro preparation of NK cells from CB require to selecte NK cells with immune-selection techniques because of low frequency [11]. In order to avoid the limitations in low number and immature state of NK cells in CB, ex vivo expansion and activation is necessary [12]. NK cells are generally isolated from CB through immunomagnetic beads selection protocols to enrich CD56-positive cells and/or deplete CD3-positive cells, and then cultured for functional expansion and activation using feeder cells, such as Epstein-Barr virus-transformed lymphoblastoid cell lines, mesenchymal stromal cells, gene-modified K562 cells expressing 4-1BB ligand and IL-15, and other irradiated tumor cell lines [5, 13]. In addition, NK cells are originally generated from CD34^+^ hematopoietic stem cells (HSCs), some studies have described an alternative method to generate NK cells with high yield, purity and functionality from CB-derived CD34^+^ HSCs under feeder cells-based conditions [10, 14–16]. Recently, a feeder cells-free method has been successfully performed for the generation of NK cells from CB-derived CD34^+^ HSCs [7, 17]. However, it needs delicate culture regimens and multiple cytokine cocktails, which may lead to high cost-effectiveness. Generally, these methods require a complicated technology of cell sorting in an initial step, and it may increase the risk of cell trauma and contamination. Furthermore, the use of feeder cells or multiple cytokines during longer-term cultures would lead to NK cell apoptosis in vivo when optimum culturing conditions are eliminated after adoptive transfer [18]. In addition, these methods are also more costly because of complex operations and supplements.

Although several methods have been proposed to generate clinically relevant NK cell products (mean: 2 × 10^9^ cells) with high purity (> 90%) from CB [13, 19], it is still difficult to obtain the sufficient numbers of highly pure NK cells from CB without cell sorting and feeder cells/multiple cytokines [13]. Previously, we had found that zoledronate could increase enrichment, expansion and activation of NK cells from CB-derived mononuclear cells (MNCs) [20]. Some studies have reported that interleukin (IL)-2 expansion could recruit and activate key regulators involved in lytic immunological synapse formation of CB-derived NK cells, enabling effective cytotoxicity against killing of acute myeloid leukemia (AML) cells in vitro and in vivo [21, 22]. Group A streptococcus preparation, which is widely used as an immunopotentiator with considerable success in patients with malignant diseases, strongly augmented human NK cell activity in vivo as well as in vitro [23]. Therefore, we try to use develop a simple method with the capability of generating NK cells with high yield, purity and functionality from CB through using zoledronate, group A streptococcus and IL-2 stimulation of MNCs without cell sorting and feeder cells/multiple cytokines.

## Results

### Preparation of NK cells from CB

**Fig. 1:**
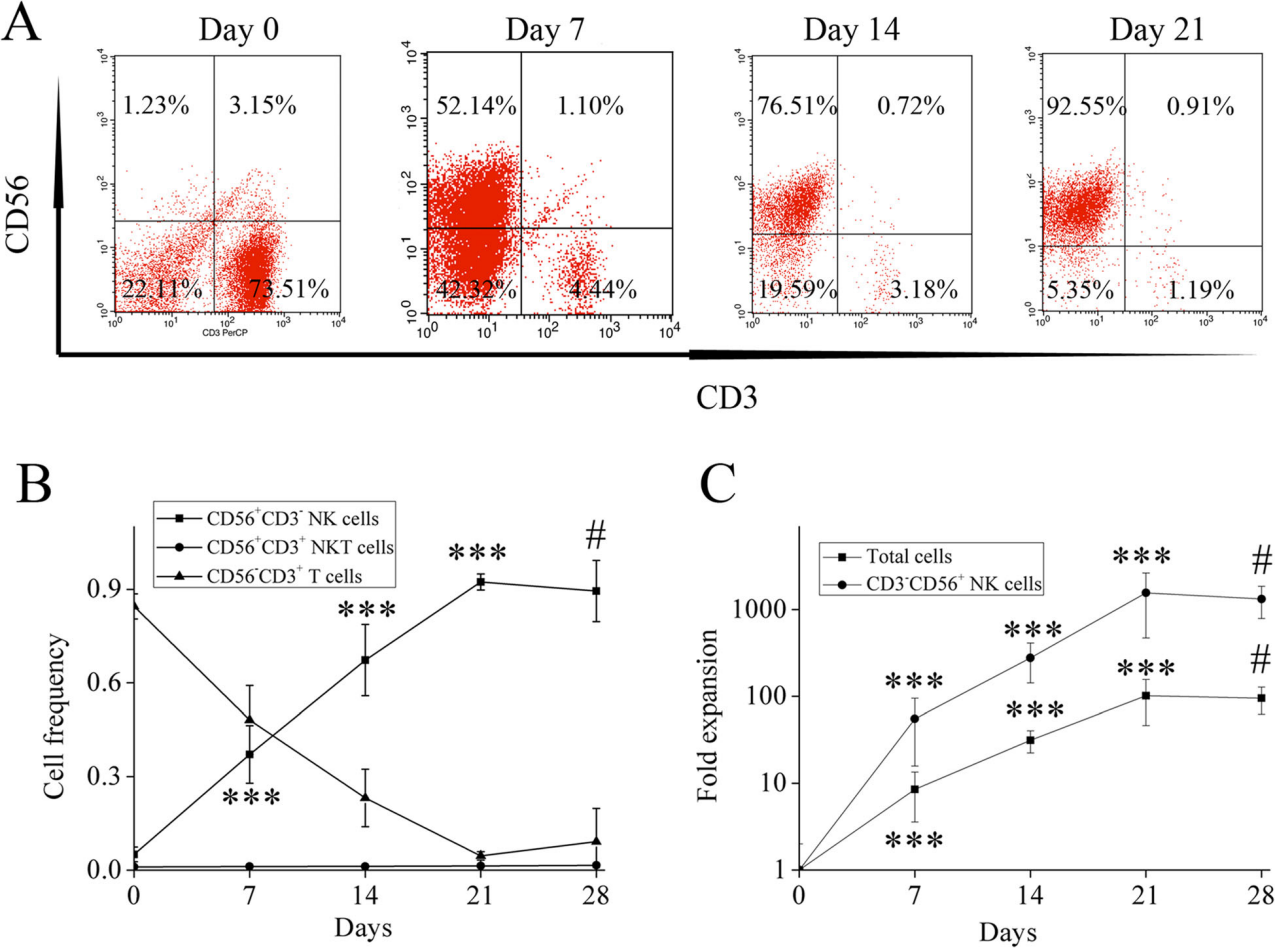
Characterization of expanded NK cells from CB. Cell proportion (CD56^+^CD3^−^ NK cell, CD56^−^CD3^+^ T cell and CD56^+^CD3^+^ NKT cell) was analyzed by flow cytometric analyses, representative FACS dot plots are presented (**a**). The kinetics of (**b**) cell proportion and (**c**) cell population (total cell and CD56^+^CD3^−^ NK cell) during culture. ***P < 0.001, indicates statistical significance increase in NK cell proportion and total and NK cell population compared to previous time points of assessment; #*P* > 0.05, indicates no statistical significance between day 28 and 21. Data are shown as mean ± standard deviation, *n* = 5

After the isolation process by Ficoll, an average of 5.02% CD56^+^CD3^−^ NK cells (range, 1.92 to 9.66%) was obtained in MNCs, whereas CD56^−^CD3^+^ T cells constituted 84.53% (range, 72.98 to 96.34%). Expansion of CD56^+^CD3^−^ NK cells was much higher compared with other types of cells, so NK cells dominated at the end of the culture, reaching on average 92.37% of the total cell populations (range: 88.91 to 96.37%; Additional file 1) by day 21. The frequency of CD56^+^CD3^+^ NKT cells remained largely unchanged before and after culture (day 0: 1.05%, day 21: 1.35%), whereas the frequency of CD56^−^CD3^+^ T cells declined, decreasing to an average of 4.56% (range: 1.30 to 9.63%; Fig. 1a and b). By day 21, the total cell count had expanded on average 101-fold (range: 65–137-fold) and, among these, CD56^+^CD3^−^ NK cell population had expanded on average 1561-fold (range: 695–2387-fold), reaching on average 1.59 × 10^10^ (range: 0.84–2.23 × 10^9^; Fig. 1c). At this time point the expansion potential reached a plateau, thus the cells reached a quiescence phase when measured later on day 28. These data demonstrate that NK cells with high yield and purity could be expanded efficiently from CB-derived MNCs ex vivo using the method described here. .

### Surface expression of NK receptors and markers

**Fig. 2:**
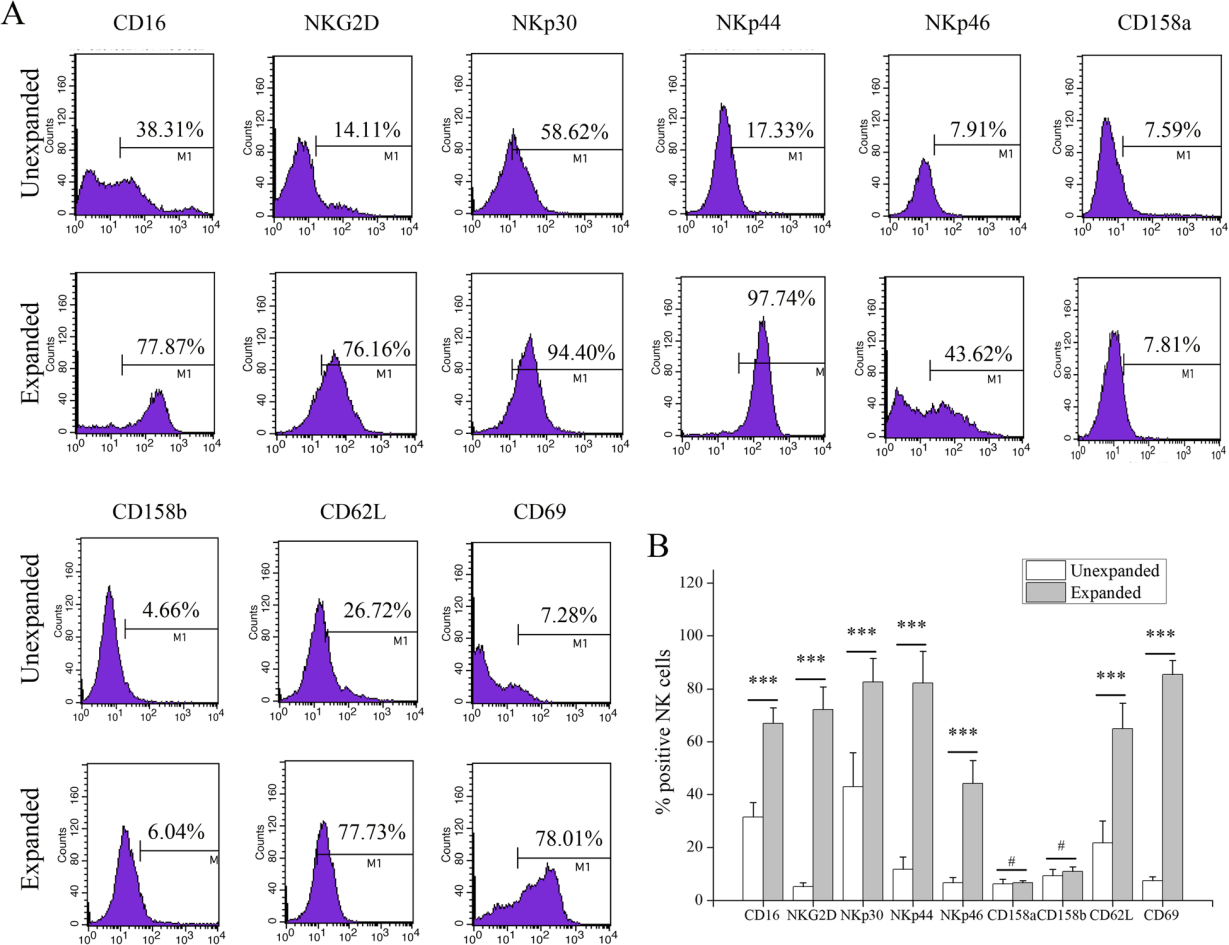
Receptors and markers of unexpanded and expanded NK cells from CB. (**a**) Representative histograms and (**b**) percentages of CD16, NKG2D, NKp30, NKp44, NKp46, CD158a, CD158b, CD62L, and CD69 expression on CD56^+^CD3^−^ NK cells of the unexpanded and expanded NK cells from CB. ***P < 0.001, indicates statistical significance increase in receptors and markers compared to unexpanded NK cells; #*P* > 0.05, indicates no statistical significance. Data are shown as mean ± standard deviation, *n* = 5

The expression of receptors and markers in expanded NK cells was assessed and compared to unexpanded NK cells. Among activating receptors, CD16 (day 0: 31.40%, day 21: 66.91%; *P* < 0.001), NKp30 (day 0: 42.99%, day 21: 82.61%; *P* < 0.001), NKp44 (day 0: 11.72%, day 21: 82.19%; *P* < 0.001), NKp46 (day 0: 6.68%, day 21: 44.20%; *P* < 0.001) and NKG2D (day 0: 5.14%, day 21: 72.20%; *P* < 0.001) significantly increased during the expansion while expression of the inhibitory receptors, CD158a (day 0: 6.33%, day 21: 6.68%; *P* > 0.05) and CD158b (day 0: 9.40%, day 21: 10.91%; *P* > 0.05) remained largely unchanged during the culture period. Also, activation markers of the NK cells including CD62L (day 0: 21.66%, day 21: 64.91%; *P* < 0.001) and CD69 (day 0: 7.47%, day 21: 85.43%; *P* < 0.001) were increased on the surface of expanded NK cells (Fig. 2). These data demonstrate that NK cells are markedly activated after expansion.

### Cytokines production of expanded NK cells

**Fig. 3:**
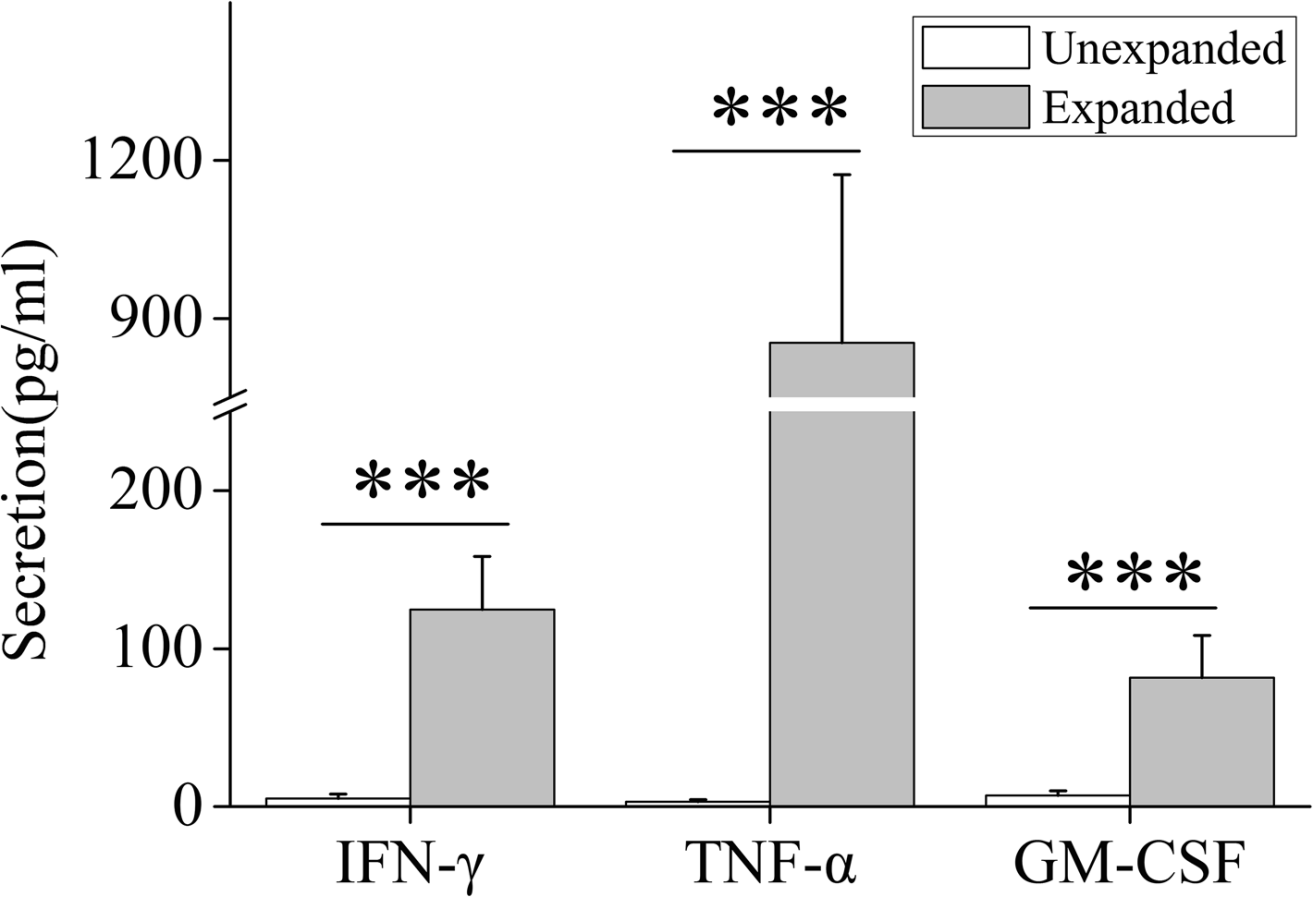
Cytokine production of unexpanded and expanded NK cells from CB. ***P < 0.001, indicates statistical significance increase in cytokine production compared to unexpanded NK cells. Data are shown as mean ± standard deviation, *n* = 5

The secretion cytokines including granulocyte-macrophage colony-stimulating factor (GM-CSF), interferon-gamma (IFN-γ), and tumor necrosis factor-alpha (TNF-α) were also assessed before and after culture. The very low level of IFN-γ (mean: 5.21 pg/mL, range: 1.37–8.45 pg/mL), TNF-α (mean: 2.99 pg/mL, range: 1.16–5.04 pg/mL) and GM-CSF (mean: 6.95 pg/mL, range: 4.38–9.81 pg/mL) secretion was observed in unexpanded NK cells. Expanded NK cells exhibited significantly increased IFN-γ (mean: 124.67 pg/mL, range: 57.95–183.12 pg/mL), TNF-α (mean: 853.94 pg/mL, range: 479.67–1201.26 pg/mL) and GM-CSF (mean: 81.67 pg/mL, range: 55.43–108.42 pg/mL) secretion compared to unexpanded NK cells (all *P* < 0.001, Fig. 3). These results demonstrate that the patterns of cytokine secretion are markedly increased after culture.

### Cytotoxicity of expanded NK cells

**Fig. 4:**
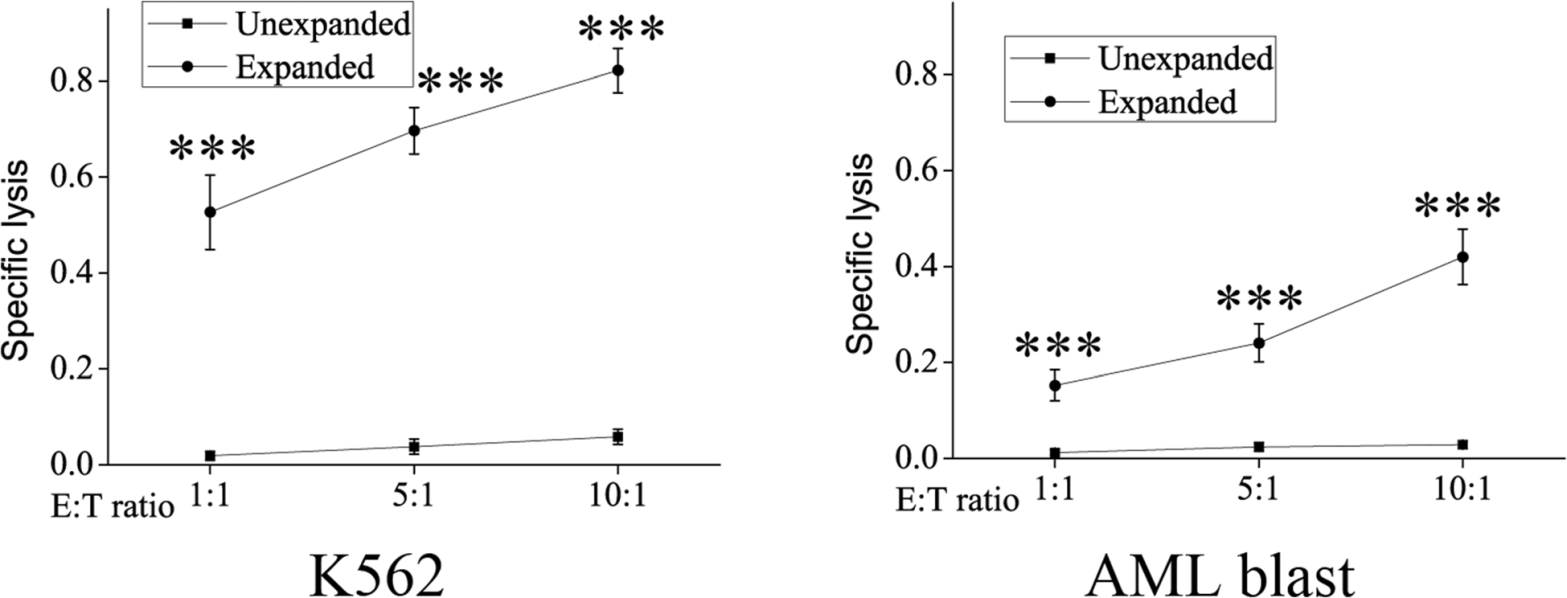
Cytolytic activities of unexpanded and expanded NK cells from CB. ***P < 0.001, indicates statistical significance increase in cytolytic activities compared to unexpanded NK cells. Data are shown as mean ± standard deviation, *n* = 5

The cytotoxicity levels of expanded vs. unexpanded NK cells against leukemia cell line (K562) and primary patient AML blasts were evaluated. The unexpanded NK cells were poor cytotoxicity against K562 cells (range: 1.91–5.86%) and primary patient AML blasts (range: 1.24–2.89%) at a variety of E:T ratios, but the expanded NK cells showed high levels of cytotoxicity against K562 (range: 52.63–82.18%) and AML targets (range: 15.28–24.07%). NK cells expanded in culture consistently exhibited a high level of cytotoxicity against K562 and AML targets compared to unexpanded NK cells at a wide range of E:T ratios (all *P* < 0.001, Fig. 4). These results show that cytotoxicity of NK cells are markedly elevated after expansion.

## Discussion

In this study, we develop a simple and economical method for in vitro preparation of NK cells through IL-2, group A streptococcus and zoledronate stimulation of CB-derived MNCs. it is a practically advantageous method that does not need cell sorting and feeder cells/multiple cytokines, which could lead to reduction in cost. Furthermore, IL-2, streptococcus A group and zoledronate have also been approved for human use, which could increase the safety. In addition, this simple method is more easily standardized and exportability. To our knowledge, this is the first report of a simple method for in vitro preparation of NK cells from CB without cell sorting and feeder cells/multiple cytokines.

Using this method, a mean number of 1.59 × 10^10^ NK cells with an average purity of 92.37% are generated after 21 days of culture. Previously, we found that zoledronate had a favorable effect on expansion of NK cells from CB-derived MNCs [20]. Furthermore, the addition of group A streptococcus induced a higher increase in the expansion fold (1561 vs. 1286) of NK cells, showing that both have the synergism. A study has reported a multiple cytokines-based delicate culture method for up to 6 weeks to generate therapeutic NK cell products (mean: 2 × 10^9^ cells) with high purity (> 90%) from CB-derived CD34^+^ HSCs [9]. Compared with this method, our method yields much higher number (8-times) of NK cells with less time. We hypothesize that this is related to very low proportion of CD34^+^ HSCs in CB and cumbersome procedures including HSCs expansion and NK cell differentiation and expansion. Other group has reported that a feeder cell-based NK cultivation method is established with the capability of generating a clinically relevant dose (mean: 1.2 × 10^9^ cells) with a purity of > 80% of CB-derived NK cells [6]. Compared with this method, our method also yields much higher number (15-times) of NK cells with the same time. A possible explanation might be that accessory cells such as monocytes in CB-derived MNCs can support NK cell expansion [24]. Therefore, A large amount of NK cells generated in vitro by our method can be, allowed for multiple infusions of NK cells.

Purity, as one of crucial release criteria for NK cell-based therapies, has not yet been standardized, but > 90% CD56^+^CD3^−^ for allogenic NK cells have been suggested in recently published study [9, 25]. Previously, we found that zoledronate had a favorable effect on enrichment of NK cells from CB-derived MNCs [20]. Furthermore, the addition of group A streptococcus induced a greater increase in the frequency (92.37% vs. 80.46%) of NK cells, showing that both have the synergism. The purity of NK cells produced by our method is comparable to cytokine-based culture method (> 90%) [9], but higher compared to feeder cell-based method (> 80%) [6].

Another crucial obstacle is the immaturity state of NK cells in CB [3]. It has been known that NK cell cytotoxicity is regulated by the complex balance between activating and inhibitory receptors. NK cells expended by our method exhibit high expression level of activating receptors CD16, NKG2D, NKp30, NKp44 and NKp46, but unchanged expression level of inhibitory receptors CD158a and CD158b compared with unexpended NK cells. This leads to high-level expression of activating markers CD62L and CD69, showing that NK cells expanded in this study have activated a cellular mechanism controlling effector function. A recent study also reported no changes in expression of CD158a and CD158b by CB-derived NK cells after incubation with IL-2, IL-12 or IL-18 [26]. This confirms that killer cell Ig-like receptor acquisition does not require group A streptococcus and zoledronate activation.

Previous studies have shown that IL-2-activated NK cells from CB exhibited significantly increased frequency of IFN-γ and TNF-α secretion compared with unexpended NK cells [21, 26]. In previous study, we showed that zoledronate increased IFN-γ, TNF-α and GM-CSF secretion by IL-2-activated NK cells from CB [20]. Early study showed that group A streptococcus stimulated human lymphocytes to produce IFN (both a- and γ-types) and IL-2 and that both factors were primarily responsible for the NK augmentation by group A streptococcus [23]. It has been previously shown that cytokines including IFN-γ, TNF-α and GM-CSF play key roles in NK cell functions [27]. To evaluate the impact of IL-2, zoledronate and group A streptococcus on the secretion of cytokines by CB NK cells, cytokines secretion was measured in the supernatants of unexpended and expanded NK cell cultures. The result shows that NK cells expanded by our method can produce robust inflammatory cytokine IFN-γ, TNF-α and GM-CSF. We further investigate that NK cells expanded by our method can induce lytic function against K562 and primary AML targets compared with unexpended NK cells, showing that ex vivo generated NK cells from CB could contribute greatly to the elimination of tumor during adoptive NK cell immunotherapy. These results are comparable to data from zoledronate only, showing no synergy between them. This might be related to MNCs rather than purified NK cells. However, in vivo functional studies are needed to explore in the next study.

## Conclusion

We have developed a simple, safe and economical method to obtain high yield, purity, and functionality NK cells from CB through IL-2, streptococcus A group and zoledronate stimulation of MNCs without cell sorting and feeder cells/ multiple cytokines. Adoptive transfer of NK cells generated from CB by this method may therefore provide a promising new paradigm for the treatment of patients with AML and other hematological malignancies. This simple method satisfies the standards for in vitro preparation of NK cells as a potential therapeutic product, is more accessible for clinical practice, and may hold valuable potential for adoptive cellular immunotherapy for hematological malignancies like AML.

## Methods

### NK cells preparation

**Fig. 5:**
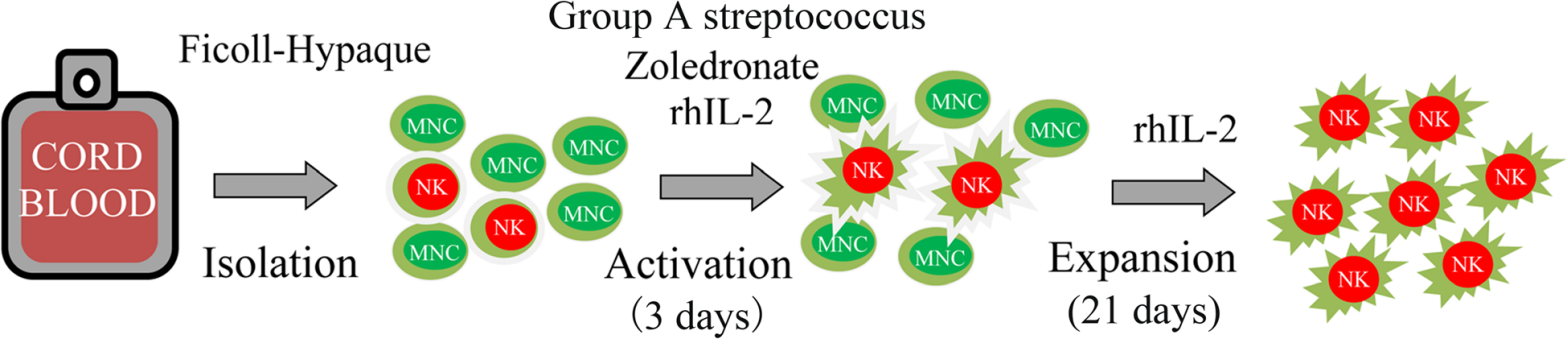
Schematic diagram of the preparation protocol for NK cells from CB

All frozen CB samples were drawn from the Shandong Cord Blood Bank. MNCs were isolated from CB using Ficoll-Hypaque density gradient centrifugation. For activation, MNCs were cultured for 3 days in T175 flasks at 2 × 10^6^ cells/mL in AIM-V serum free media (Life Technologies) supplemented with 2000 IU IL-2/mL (Four Rings Biopharma, China), 0.01KE/mL group A streptococcus (Lu Ya Pharma, China) and 5 μM zoledronate (Novartis Pharma). For expansion, the fresh medium containing 2000 IU IL-2/mL was added every 2 to 3 days for 21 days. An outline of the preparation protocol is summarized in Fig. 5.

### Expansion kinetics assessment

Total cell counts were determined by trypan blue staining on days 0, 7, 14, 21 and 28. Absolute cell numbers were calculated by multiplying the total cell count by the percentage of each cell type determined by flow cytometry. Cell expansion efficiency was expressed as “fold expansion” which was determined by dividing the number of total cells or absolute NK cells on days 7, 14, 21 and 28 by the number on day 0.

### Flow Cytometr

The cells were labeled with fluorochrome-conjugated mouse mAbs against human cluster of differentiation CD3, CD56, CD16, CD158a, CD158b and NKG2D (BD Biosciences); NKp30, NKp44 and NKp46 (Beckman Coulter); CD69, CD62L (Miltenyi Biotec). Isotype-matched antibodies were used as controls. Flow cytometry analysis was performed using a MACSQuant Analyzer (Miltenyi Biotec). Data were analyzed with MACSQuantify Software.

### Cytokines release assay

The cell culture supernatants were collected, the level of cytokines including IFN-γ, TNF-α and GM-CSF in supernatants was analyzed using enzyme-linked immunosorbent assay kits (eBioscience, Inc) according to the manufacturer’s instructions.

### Cytotoxicity assay

The cytotoxicity of NK cells was analyzed using the standard 4-h ^51^Cr release assay. The NK-sensitive leukemia cell line (K562) and primary AML blasts (target) were labeled with ^51^Cr sodium chromate and incubated with NK cells (effector) at different target-to-effector (E:T) ratios (1:1, 5:1, and 10:1) in U-bottomed 96-well plates. ^51^Cr-release was determined in the supernatant after co-culture for 4 h. The maximum release was measured by treating target cells with 2% Triton X-100 and spontaneous release was determined with medium alone. The specific lysis was calculated according to the formula: (experimental release - spontaneous release) / (maximum release - spontaneous release).

### Statistical analysis

Data are presented as the mean ± standard deviation. Statistical difference was determined using a student’s t test when comparing two groups, or a one-way ANOVA analysis when more than two groups (Graphpad Prism 5.0). *P* < 0.05 was considered significant.

## Supplementary information

**Additional file 1: Figure S1.** FACS analysis gating strategy for NK cells expanded on day 21.

## Data Availability

The datasets used and analyzed during the current study are available from the corresponding author on reasonable request.

## Abbreviations

AML: Acute myeloid leukemia
CB: Cord blood
E:T: Effector-to-target
GM-CSF: Granulocyte-macrophage colony-stimulating factor
HSCs: Hematopoietic stem cells
IFN-γ: Interferon-gamma
IL: Interleukin
MNCs: Mononuclear cells
NK: Natural killer
TNF-α: Tumor necrosis factor-alpha
