# Supplementary figures and images for: A simple method for in vitro preparation of natural killer cells from cord blood
Source: BMC Biotechnol. 2019 Nov 21;19:80. doi: 10.1186/s12896-019-0564-0 (PMC6869212; doi:10.1186/s12896-019-0564-0)

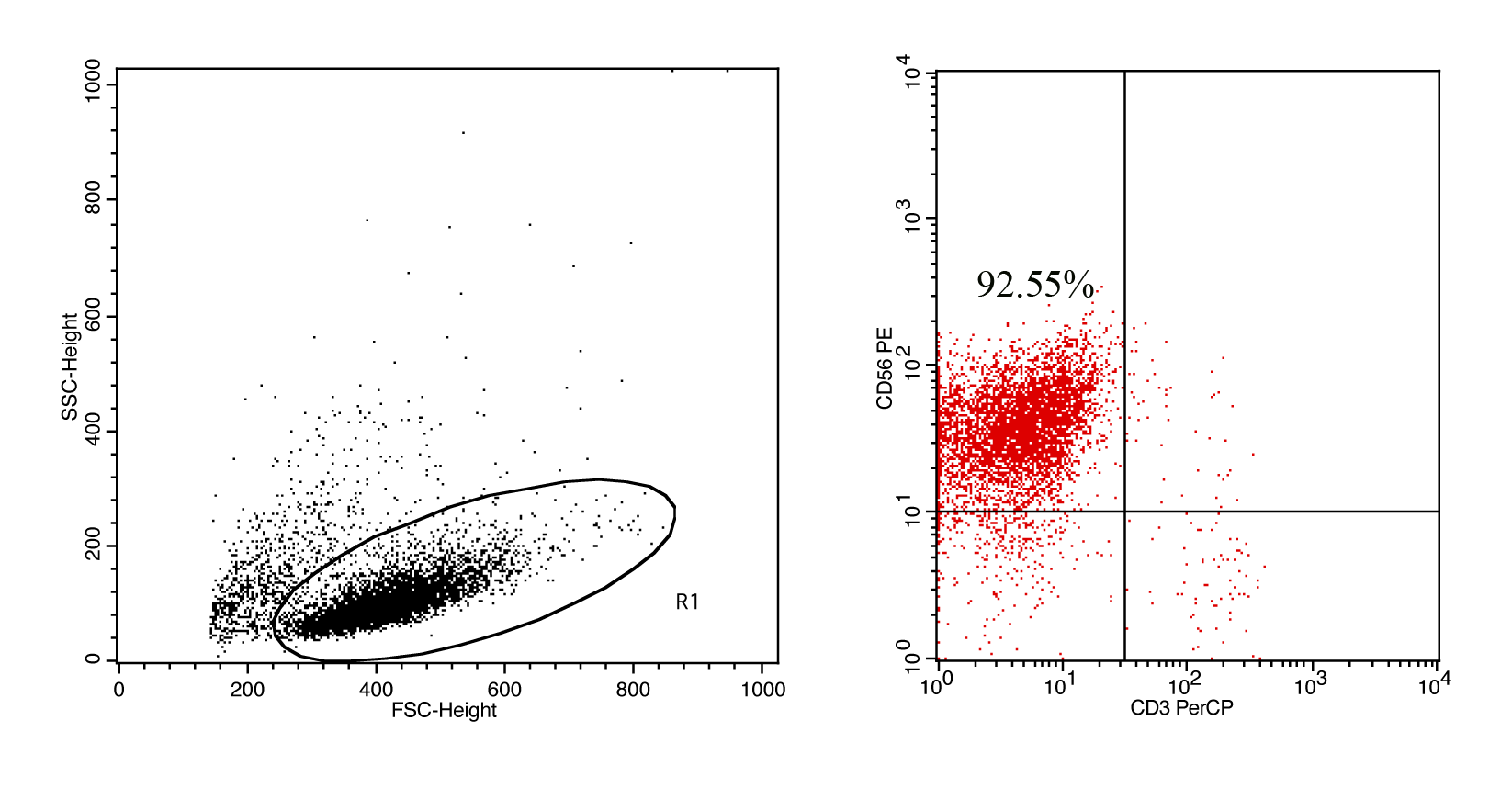

Supplement: Supplementary file 1 — Additional file 1: Figure S1. FACS analysis gating strategy for NK cells expanded on day 21. [file 12896_2019_564_MOESM1_ESM.tif]
